# Cavin4b/Murcb Is Required for Skeletal Muscle Development and Function in Zebrafish

**DOI:** 10.1371/journal.pgen.1006099

**Published:** 2016-06-13

**Authors:** Michael P. Housley, Brian Njaine, Filomena Ricciardi, Oliver A. Stone, Soraya Hölper, Marcus Krüger, Sawa Kostin, Didier Y. R. Stainier

**Affiliations:** 1 Department of Biochemistry and Biophysics, University of California, San Francisco (UCSF), San Francisco, California, United States of America; 2 Max Planck Institute for Heart and Lung Research, Bad Nauheim, Germany; University of Pennsylvania School of Medicine, UNITED STATES

## Abstract

Skeletal muscles provide metazoans with the ability to feed, reproduce and avoid predators. In humans, a heterogeneous group of genetic diseases, termed muscular dystrophies (MD), lead to skeletal muscle dysfunction. Mutations in the gene encoding Caveolin-3, a principal component of the membrane micro-domains known as caveolae, cause defects in muscle maintenance and function; however it remains unclear how caveolae dysfunction underlies MD pathology. The Cavin family of caveolar proteins can form membrane remodeling oligomers and thus may also impact skeletal muscle function. Changes in the distribution and function of Cavin4/Murc, which is predominantly expressed in striated muscles, have been reported to alter caveolae structure through interaction with Caveolin-3. Here, we report the generation and phenotypic analysis of *murcb* mutant zebrafish, which display impaired swimming capacity, skeletal muscle fibrosis and T-tubule abnormalities during development. To understand the mechanistic importance of Murc loss of function, we assessed Caveolin-1 and 3 localization and found it to be abnormal. We further identified an *in vivo* function for Murc in Erk signaling. These data link Murc with developmental defects in T-tubule formation and progressive muscle dysfunction, thereby providing a new candidate for the etiology of muscular dystrophy.

## Author Summary

Membrane structures are critical to skeletal muscle excitation-contraction coupling, and disruptions in key membrane proteins can lead to muscular dystrophy. Caveolae, micro-domains in the cell membrane, are known to be important in forming muscle-specific membrane structures such as the T-tubule network and play a role in some forms of muscular dystrophy. Two classes of proteins help form caveolae: Caveolins and Cavins. However, it is poorly understood how Cavins function in skeletal muscle development or whether Cavin dysfunction can lead to muscular dystrophy. To address these questions, we generated a Cavin4b/Murcb deficient zebrafish line by targeted TALEN mutagenesis and found that Cavin4b/Murcb is necessary for the formation and function of skeletal muscle. Cavin4b/Murcb mutant zebrafish exhibit progressive skeletal muscle deterioration, disrupted membrane structures early in development and aberrant growth factor signaling. Cavin4b/Murcb deficient fish have smaller and irregularly shaped muscle fibers with fibrosis, similar to what is observed in muscular dystrophy. Cavin4b/Murcb mutant fish also exhibit mislocalized Caveolin-1 and Caveolin-3 as well as disrupted T-tubules, suggesting that Cavin4b/Murcb regulates the distribution of caveolar proteins and that this misregulation impacts the development of the T-tubule network.

## Introduction

Muscular dystrophies (MD) are a heterogeneous group of genetic diseases that result in progressive dysfunction of skeletal muscle [1]. Muscle weakness and loss of muscle mass can begin in childhood, while some forms of the disease manifest in adulthood. Different groups of muscles are affected depending on the type of MD and complications can include confinement to a wheelchair, scoliosis, difficulty swallowing, impaired breathing, cardiomyopathies, and early death [2,3]. Although MD can be caused by mutations in more than 30 different genes [4], common phenotypic characteristics include disruption in the membrane network [5] and abnormal calcium flux in skeletal muscle [6,7]. Loss of membrane integrity has been shown by the intracellular accumulation of proteins normally absent in skeletal muscle [8,9]. Membrane stability requires functional costameres [10], which link intracellular force generation with the extracellular matrix [11,12] via the dystrophin glycoprotein complex [13].

Cell membrane (sarcolemma) stability is critical to muscle cells, which must maintain a resting membrane potential in order to propagate action potentials via voltage-gated ion channels [14]. Excitation-contraction coupling requires specialized membrane structures, the transverse (T)-tubules and terminal cisternae, for calcium flux to trigger an actin/myosin dependent contraction. Two terminal cisternae are found on opposing sides of the T-tubules and form a structure known as the triad [15]. Disruption of calcium signaling also has long term consequences for skeletal muscle health by altering signaling pathways required for muscle remodeling [16] and may underlie the necrosis seen in MD [6]. Interestingly, in a zebrafish model of Duchenne muscular dystrophy, a serotonin reuptake inhibitor was shown to improve skeletal muscle health through improved calcium homeostasis [17].

In mouse, the development of the T-tubule network begins with invaginations in the sarcoplasmic reticulum at embryonic day (E)15 followed by postnatal maturation of the network [15]. Thus far, a few proteins are known to be required for T-tubulogenesis: MTM1, JPH1, MG29, DYSF, BIN1, and Caveolin-3 (CAV3). Loss of MTM1 in humans results in a disease known as myotubular myopathy and is characterized by severe muscle atrophy at birth, and mice lacking MTM1 show T-tubule disorganization with disrupted calcium homeostasis and excitation-contraction coupling defects [18]. Bin1, which possesses membrane deforming properties, has been shown to be enriched at T-tubules and induce tubular invaginations in the plasma membrane [19]. Loss of CAV3 can result in several skeletal muscle diseases including type 1C limb-girdle muscular dystrophy [20]. Knockout of *Cav3* in mouse leads to disrupted dystrophin glycoprotein complex distribution as well as abnormal T-tubulogenesis [21], and Cav3 is necessary for skeletal muscle differentiation *in vitro* [22,23] and in zebrafish [24].

Primarily expressed in striated muscle, Cav3 is an integral membrane protein required for the formation of caveolae, invaginations in the cell membrane that function in lipid storage, signaling and endocytosis [25]. Caveolin-1 (Cav1), a broadly expressed member of the Caveolin family, is required for lipid homeostasis and insulin sensitivity [26]. The Caveolin proteins interact with a class of membrane-associated proteins termed Cavins, which are enriched in caveolae and are critical for their formation. The Cavin family consists of 4 members, one of which is Cavin4, also known as Murc (Muscle-restricted coiled-coil protein).

*Cavin4/Murc* was originally discovered through SAGE screening of a cDNA library prepared from adult mouse hearts [27] and expression analysis revealed high mRNA expression in both heart and skeletal muscles. In cultured C2C12 myoblasts, CAVIN4/MURC expression was found to increase during differentiation [28]. Additionally, shRNA-mediated knockdown of CAVIN4/MURC resulted in the inhibition of myotube formation. Concurrent with these initial studies, *Cavin4/Murc* was also identified by scanning the genome for homologs of three known Cavin genes and was thus termed “*Cavin4*" [29]. The four members of the Cavin family were found to localize to the cell membrane in a CAV1-dependent manner, and in skeletal muscle, CAVIN4/MURC co-localizes with CAV3 in putative caveolae [29]. In cardiomyocytes, CAVIN4/MURC localizes to caveolae and T-tubules and its over-expression results in disrupted caveolar shape [30]. Furthermore, in a patient with rippling muscle disease and mosaically expressed CAV3, muscle fibers lacking CAV3 had a distinct absence of CAVIN4/MURC expression [29].

The Cavin proteins possess substantial homo- and hetero-oligomerization capacity via two helical repeat (HR) domains [31]. Although their functional importance for caveolar formation is unclear, Cavins have the capacity to remodel membranes and it has been proposed that they polymerize to form large assemblies of rod-like structures stabilized by interactions with membranes [31].

Here, we report the generation of a zebrafish line carrying a TALEN-induced *murcb* mutant allele and show that Cavin4b/Murcb is necessary for skeletal muscle function in both larval and adult fish. Cavin4b/Murcb deficiency results in progressive dysfunction and fibrosis of skeletal muscles. Additionally, we show that disruption of Cavin4b/Murcb is associated with changes in Erk signaling and Caveolin expression in adult zebrafish. Larvae lacking Cavin4b/Murcb exhibit an increased number of caveolae, mislocalization of Caveolin-1 expression, and T-tubule defects.

## Results

### Cavin4/Murc is conserved between zebrafish and mammals and is expressed in the somites during zebrafish development

**Fig 1:**
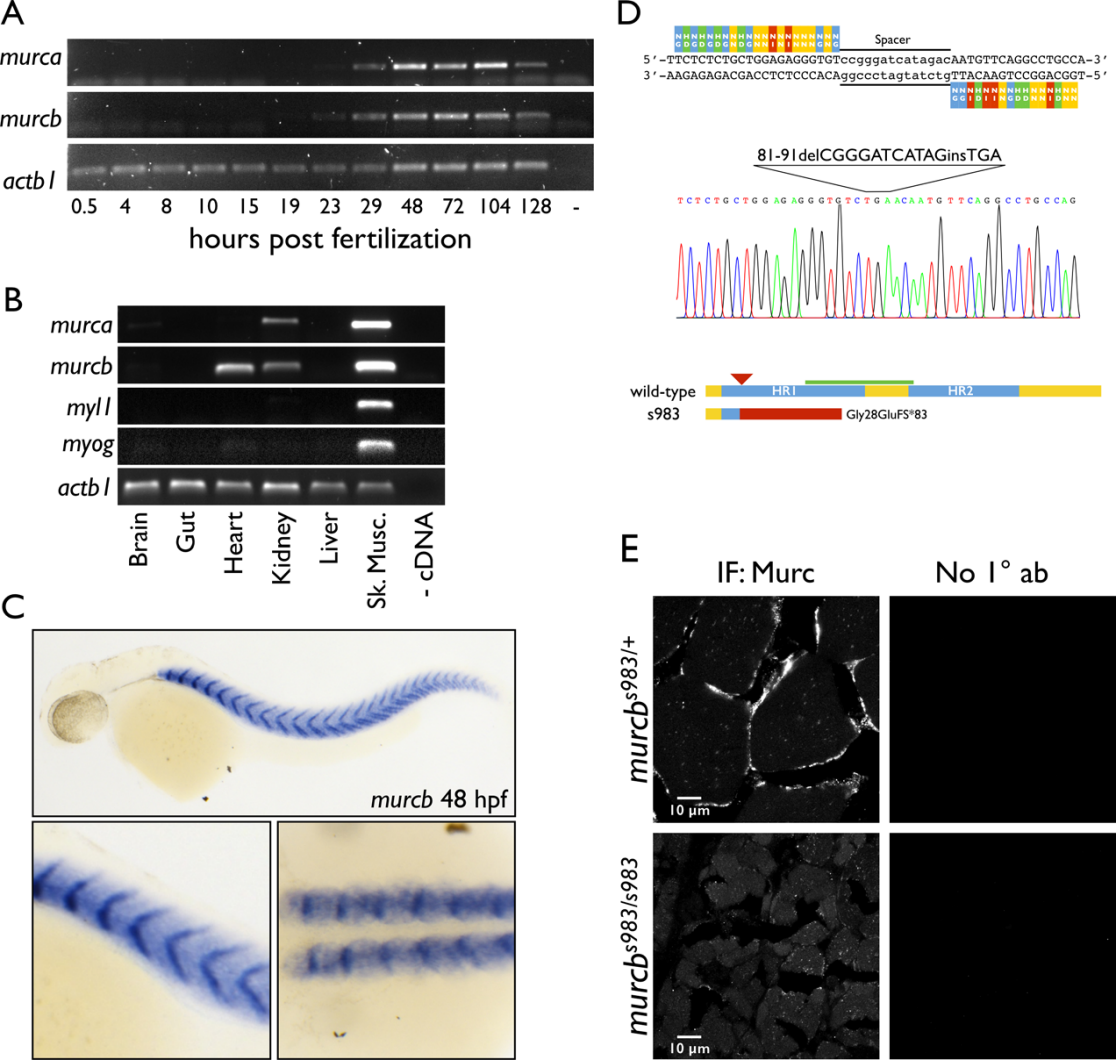
TALEN design and generation of a *murcb* mutant allele. A. RT-PCR analysis of mRNA expression of *murca and murcb* during zebrafish development. *actb1* is shown as a loading control. B. RT-PCR analysis of mRNA expression of *murca* and *murcb* in adult zebrafish tissues. *myl1* and *myog* are shown as controls for skeletal muscle contamination of other tissue samples. *actb1* is shown as a loading control. C. *Whole mount in situ* hybridization of *murcb* mRNA in zebrafish embryos at 48 hpf. Top: lateral view. Bottom left: magnified lateral view. Bottom right: magnified dorsal view. D. TALEN construct for *murcb* mutagenesis. Sequencing results for the *murcb*^*s983*^ allele. Schematic of wild-type and predicted mutant proteins. The *murcb*^*s983*^ lesion leads to a premature stop codon after 83 missense amino acids starting at amino acid 28. Helical region (HR) domains are indicated in blue. The red arrowhead points to the TALEN target site. The red bar indicates the region of the mutant protein that is out of frame. The green bar indicates the antigen used to generate the antibody used in this work. E. Anti-Cavin4/Murc immunofluorescence (IF) micrographs of 10 μm transverse sections of skeletal muscle prepared from 10 wpf sibling and *murcb*^*s983/s983*^ fish. No 1° ab indicates control samples incubated only with secondary antibodies.

Using a combinatorial Translating Ribosome Affinity Purification (TRAP) approach, we previously found that *murca* and *murcb* are expressed in the somites of developing zebrafish [32]. To understand the function of the *murc* genes, we first examined their conservation and spatiotemporal expression pattern. We aligned the protein sequences of Cavin4a/Murca and Cavin4b/Murcb with mammalian and *Xenopus* paralogs (S1A Fig) and concluded that *murca* and *murcb* are indeed duplicated genes by examining their genomic structure (S1B Fig) and through phylogenetic analysis of the protein sequences (S1C Fig). To determine when *murca* and *murcb* are expressed, we performed RT-PCR analysis on RNA isolated from developing zebrafish embryos and larvae at various times and found that mRNA for both *murc* paralogs are detectable beginning around 29 hours post fertilization (hpf) and expressed through 128 hpf (Fig 1A). In adult tissues, we found *murca* and *murcb* to be highly expressed in skeletal muscle, and to a reduced extent, both *murc* paralogs are also expressed in the kidney (Fig 1B). Interestingly, *murcb*, but not *murca*, is expressed in the adult zebrafish heart. To determine the spatial distribution of *murca* and *murcb* in zebrafish larvae, we performed *in situ* hybridization at 48 hpf and found expression in the somites with a higher signal at the somite boundaries (Figs 1C and S1D).

### Generation of a *murcb* mutant allele through TALEN mutagenesis

To test the function of *murcb in vivo*, we generated a mutant allele with TALE-nucleases designed to cut in the first exon. We used the TALEN Targeter 2.0 software from the Bogdanove laboratory to design the constructs and the Golden Gate cloning kit from Bogdanove and Voytas to generate the constructs from which mRNA was made and injected into one-cell stage embryos (Fig 1D) [33,34]. An allele of *murcb* with an 11 base pair deletion and 3 base pair insertion was identified and designated *s983*. The putative protein product encoded by the *s983* allele includes a frameshift in the 28th codon (p.G28QfsX83) and a premature stop in the 110th codon. The mutant protein is predicted to lack the conserved HR1 and HR2 domains that mediate interactions with the Cavin proteins [31]. To determine whether the *s983* lesion results in altered Cavin4b/Murcb protein expression, we performed antibody staining on transverse sections of skeletal muscle harvested from 10 wpf zebrafish and found a reduction in anti-Cavin4/Murc immunoreactivity (Fig 1E). To confirm that the *s983* homozygous mutant larvae lack Cavin4b/Murcb, we performed tandem mass spectrometry and found that peptides corresponding to Cavin4b/Murcb were not detected in lysates from mutant larvae but were clearly present in heterozygous controls (S1 Table, S2 Fig). Altogether, these data indicate that *s983* is a severe mutant allele.

### Cavin4b/Murcb deficient zebrafish are smaller and have defects in skeletal muscle morphology

**Fig 2:**
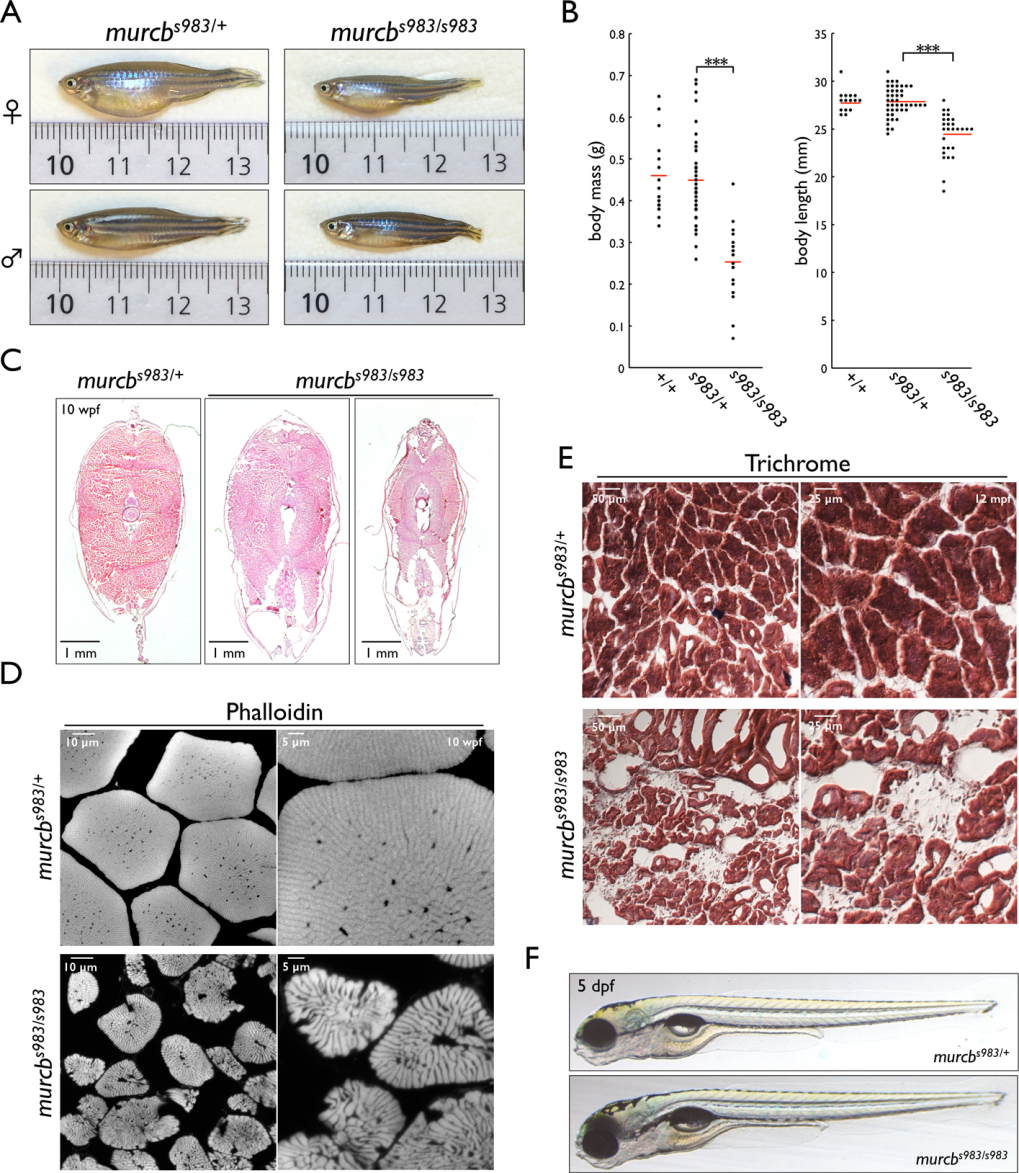
Characterization of Cavin4b/Murcb deficient zebrafish. A. Representative lateral views of *murcb*^*s983/s983*^ and *murcb*^*s983/+*^ sibling zebrafish at 10 wpf. Fish in the top panels are females while those in the bottom panels are males. B. Dot plots of body mass and length of 10 wpf wt, *murcb*^*s983/+*^, and *murcb*^*s983/s983*^ zebrafish. Length measurements were made from the anterior point to the caudal fin/trunk boundary. Approximately equal numbers of male and female fish were used. The red line represents the mean. ***p<0.0005 C. H&E stain of 10 μm sections prepared from the trunk of 10 wpf *murcb*^*s983/+*^ and *murcb*^*s983/s983*^ zebrafish. Two examples of *murcb*^*s983/s983*^ fish of different sizes are shown. D. Phalloidin staining of 10 μm transverse sections prepared from the trunk of 10 wpf *murcb*^*s983/+*^ and *murcb*^*s983/s983*^ zebrafish. E. Trichrome staining of 10 μm transverse sections prepared from the trunk of 12 mpf *murcb*^*s983/+*^ and *murcb*^*s983/s983*^ zebrafish. F. Representative lateral views of *murcb*^*s983/+*^ and *murcb*^*s983/s983*^ zebrafish at 5 dpf.

To characterize the phenotype of *murcb* mutant zebrafish, we examined 10 wpf mutant and siblings from in-crosses of *murcb*^*s983/+*^ animals and measured body mass and length and found that *murcb*^*s983/s983*^ fish were smaller with an average mass of 0.25 g compared to 0.46 g for *murcb*^*+/+*^ fish and 0.45 g for *murcb*^*s983/+*^ fish (Fig 2A and 2B). The average length of *murcb*^*s983/s983*^ animals at 24.4 mm also differed significantly from the *murcb* wild-type and heterozygous animals with average lengths of 27.7 mm and 27.9 mm, respectively.

To analyze skeletal muscle structure in *murcb* mutant fish, we harvested trunk tissue from animals sacrificed at 10 wpf and examined transverse sections histologically. Hematoxylin and eosin (H&E) staining revealed that the overall organization and symmetry of the trunk was not altered in *murcb*^*s983/s983*^ fish (Fig 2C) compared with sibling controls, though skeletal muscle fibers appeared smaller. F-actin visualization in these transverse sections revealed that skeletal muscle fibers were smaller and less uniform in size and shape in *murcb*^*s983/s983*^ fish compared to heterozygous siblings (Fig 2D). To assess the skeletal muscle pathology of Cavin4b/Murcb deficient zebrafish, we performed trichrome staining of transverse sections made from 12 mpf trunk tissue and found evidence of fibrosis in mutant animals but not in sibling controls (Fig 2E).

**Fig 3:**
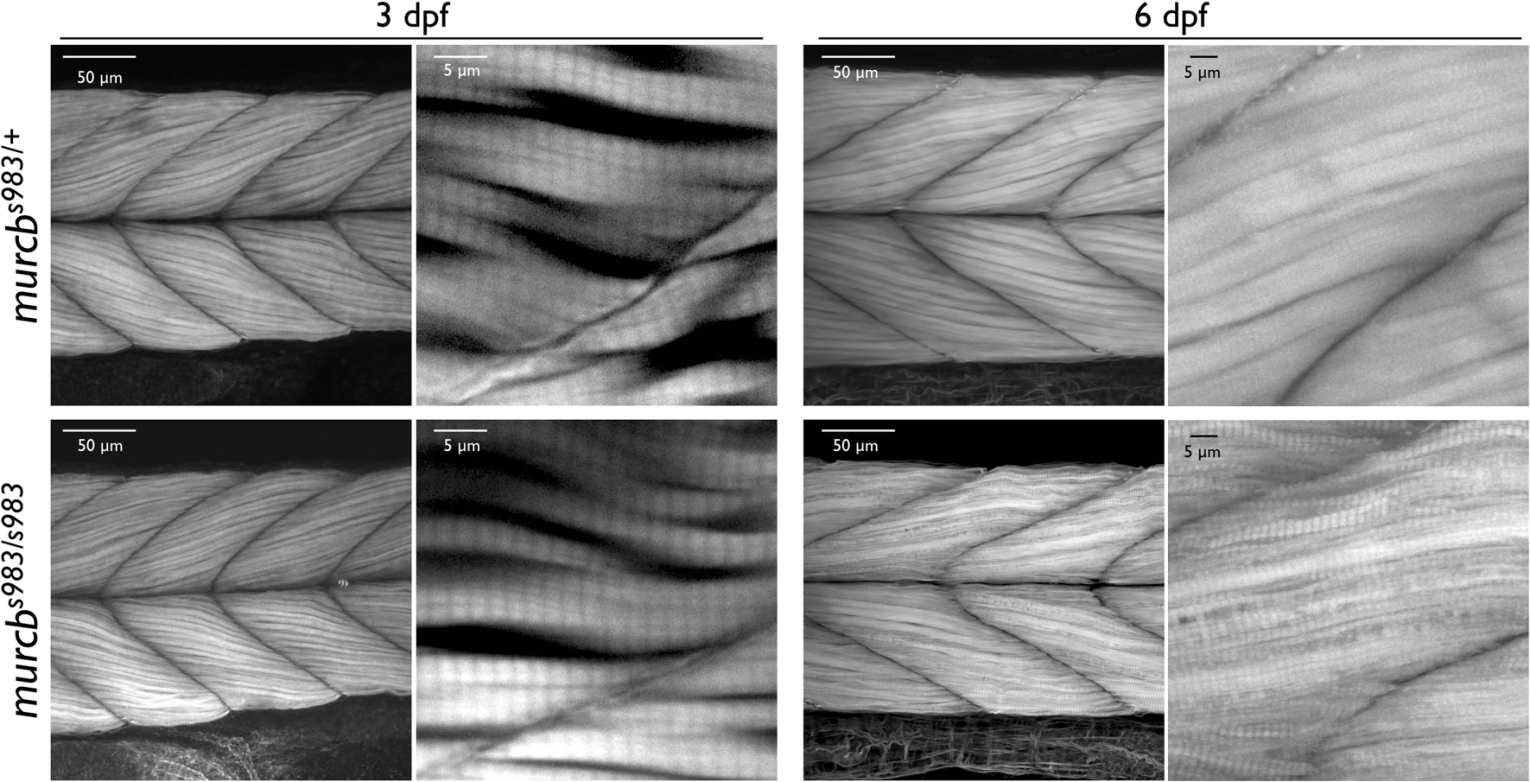
Filamentous actin analysis of Cavin4b/Murcb deficient zebrafish. Representative lateral views of confocal projections of phalloidin stained *murcb*^*s983/+*^ and *murcb*^*s983/s983*^ zebrafish at 3 dpf and 6 dpf. Skeletal muscle fibers are smaller and less consistent in size in *murcb* mutant animals compared to wild-type siblings.

To determine whether there is a role for Cavin4b/Murcb during the early development of skeletal muscle structure, we initially examined *murcb*^*s983/s983*^ larvae at 5 dpf and found no gross morphological differences with *murcb*^*s983/+*^ siblings (Fig 2F). Additionally, whole mount phalloidin staining of skeletal muscle F-actin revealed no obvious differences between *murcb*^*s983/s983*^ larvae and *murcb*^*s983/+*^ siblings (Fig 3) at 3 dpf. In contrast, at 6 dpf we found that skeletal muscle fibers were smaller and less consistent in size in *murcb*^*s983/s983*^ larvae compared with *murcb*^*s983/+*^ siblings (Fig 3). To determine whether the membranes of the skeletal muscle were intact in *murcb* mutant larvae, we performed Evans blue dye staining [35], and found the somites of both heterozygous *murcb*^*s983/+*^ and *murcb*^*s983/s983*^ larvae to be impermeable to the dye (S3A Fig). In order to understand whether apoptosis was contributing to the disruption in skeletal muscle fiber size in *murcb*^*s983/s983*^ larvae, we performed acridine orange staining on whole mount zebrafish and found no difference between mutants and *murcb*^*s983/+*^ siblings (S3B Fig). Additionally, we assessed mRNA stability for both *murca* and *murcb* by RT-PCR in both *murcb*^*s983/+*^ and *murcb*^*s983/s983*^ larvae and found that transcripts for both *murc* paralogs were relatively unaffected (S3C Fig).

### Cavin4b/Murcb deficient fish have reduced skeletal muscle function

**Fig 4:**
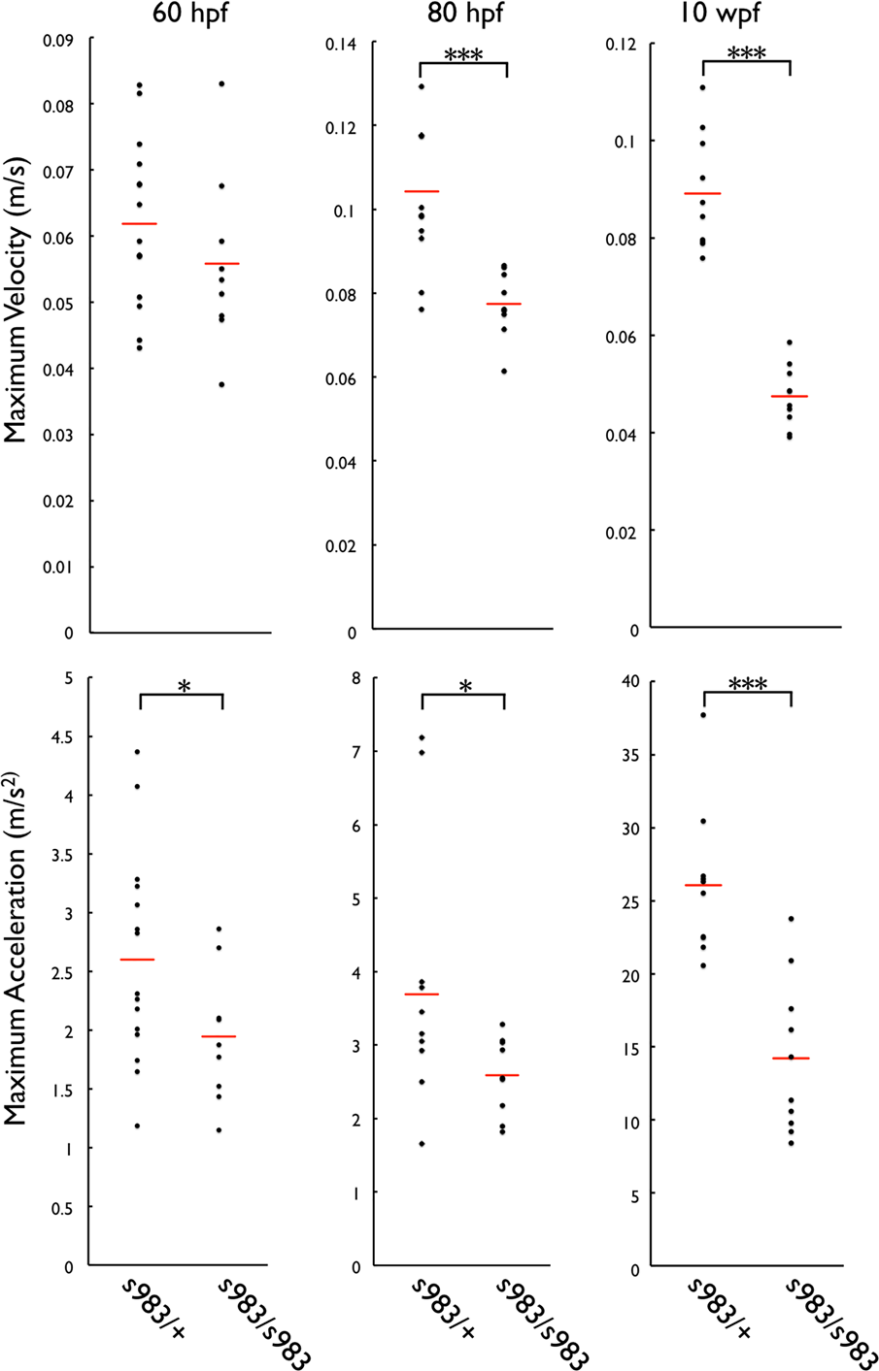
Swimming analysis of Cavin4b/Murcb deficient zebrafish. Dot plots of maximum swimming velocity and acceleration following the startle response of 60 hpf, 80 hpf, and 10 wpf *murcb*^*s983/+*^ and *murcb*^*s983/s983*^ zebrafish. The red line represents the mean. *p<0.05 ***p<0.0005

To determine whether structural defects in the skeletal muscle of *murcb* mutants affect skeletal muscle function, we quantified the maximum swimming velocity and acceleration following the startle response. At 10 wpf, *murcb*^*s983/s983*^ fish were significantly slower with a maximum velocity of 0.473 m/s compared to their heterozygous siblings that had a maximum velocity of 0.891 m/s (Fig 4). Acceleration was also reduced in *murcb*^*s983/s983*^ fish at 14.2 m/s^2^ compared with 26.1 m/s^2^ for heterozygous siblings.

In order to determine whether there was a functional defect in Cavin4b/Murcb deficient skeletal muscle during development, we measured the maximum velocity and acceleration at 60 and 80 hpf. We found that at 60 hpf, mean maximum swimming velocity was not significantly different between *murcb*^*s983/+*^ and *murcb*^*s983/s983*^ embryos (Fig 4, 0.062 *versus* 0.056 m/s, respectively); however, mean maximum acceleration was reduced in *murcb*^*s983/s983*^ embryos (1.9 m/s^2^) compared to heterozygous siblings (2.6 m/s^2^). At 80 hpf, the difference in maximum velocity between *murcb*^*s983/+*^ and *murcb*^*s983/s983*^ larvae was significantly different (0.104 *versus* 0.077 m/s, respectively). The difference in maximum acceleration between *murcb*^*s983/+*^ and *murcb*^*s983/s983*^ larvae was also significantly different (3.69 *versus* 2.59 m/s^2^, respectively). To rule out an effect of loss of Cavin4b/Murcb on the neuromuscular junctions, we performed whole mount α-bungarotoxin and Synaptic Vesicle glycoprotein 2 staining at 80 hpf. No obvious differences in post-synaptic structures could be observed between *murcb*^*s983/+*^ and *murcb*^*s983/s983*^ larvae (S4 Fig).

### Dysregulation in voltage-gated ion channel mRNA expression and T-tubule defects in Cavin4b/Murcb deficient zebrafish

**Fig 5:**
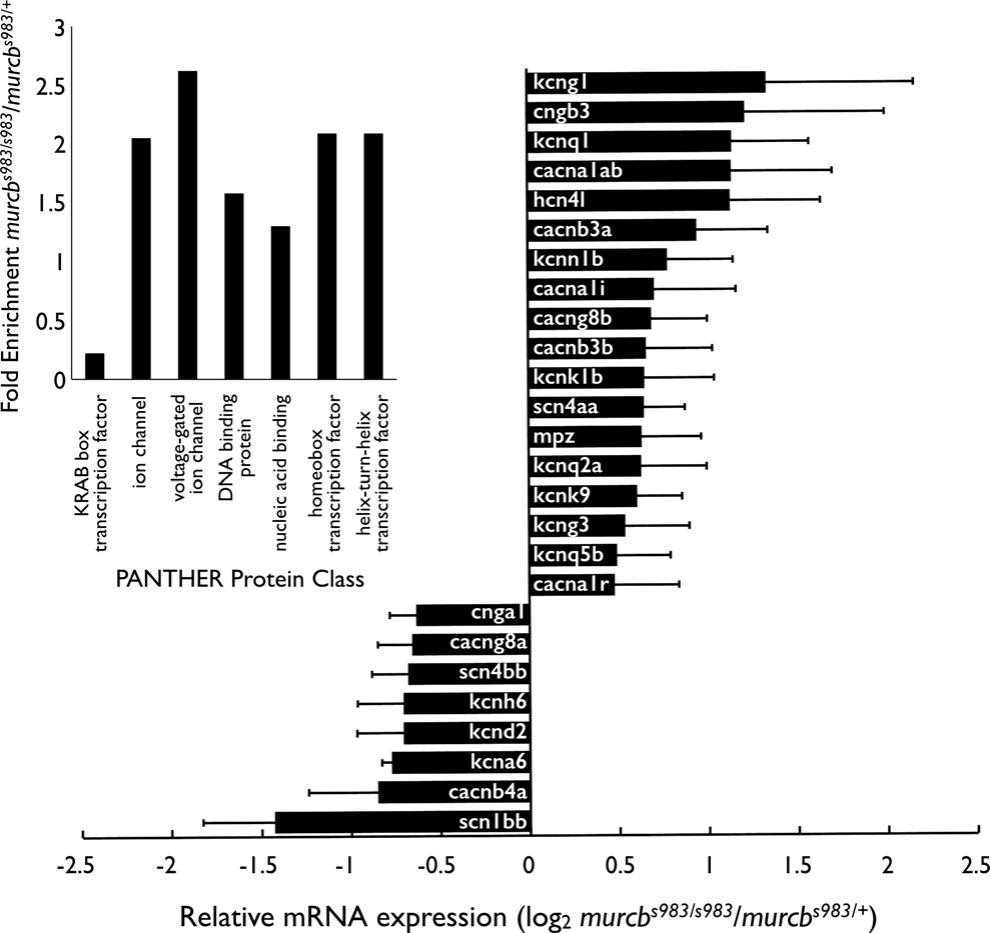
Transcriptional profiling of Cavin4b/Murcb deficient zebrafish. Inset: PANTHER Database protein class statistical overrepresentation test on microarray mRNA expression profiling from *murcb*^*s983/s983*^ compared to *murcb*^*s983/+*^ 72 hpf larvae. 1309 genes with greater than 1.5 fold or less than 0.7 fold expression difference in Cavin4b/Murcb deficient larvae compared to their heterozygous siblings were recognized in the database with 25708 genes comprising the *Danio rerio* reference list. Protein classes shown had a *p* value <0.05. The log_2_ ratio of *murcb*^*s983/s983*^/*murcb*^*s983/+*^ normalized microarray values from genes in the voltage-gated ion channel PANTHER protein class show that 26 members of the voltage-gated ion channel class were misregulated where 10 would be expected. Error bars represent SEM.

To investigate the underlying cause of the swimming defect in Cavin4b/Murcb deficient larvae, we isolated mRNA from mutant and sibling zebrafish at 72 hpf for microarray analysis. We examined the list of genes with an expression ratio of greater than 1.5 or less than 0.7 in *murcb*^*s983/s983*^ compared to *murcb*^*s983/+*^ samples by the PANTHER database protein class statistical overrepresentation test. 1309 transcripts were differentially expressed and recognized in the PANTHER database. Of these 55 are annotated in the ion channel protein class and 26 are annotated as voltage-gated ion channels while the expected number from 1309 input transcripts is 27 (*p* = 0.000177) and 10 (*p* = 0.00291), respectively (Fig 5). The relative mRNA expression levels of the differentially expressed genes in the voltage-gated ion channel class are also shown (Fig 5).

**Fig 6:**
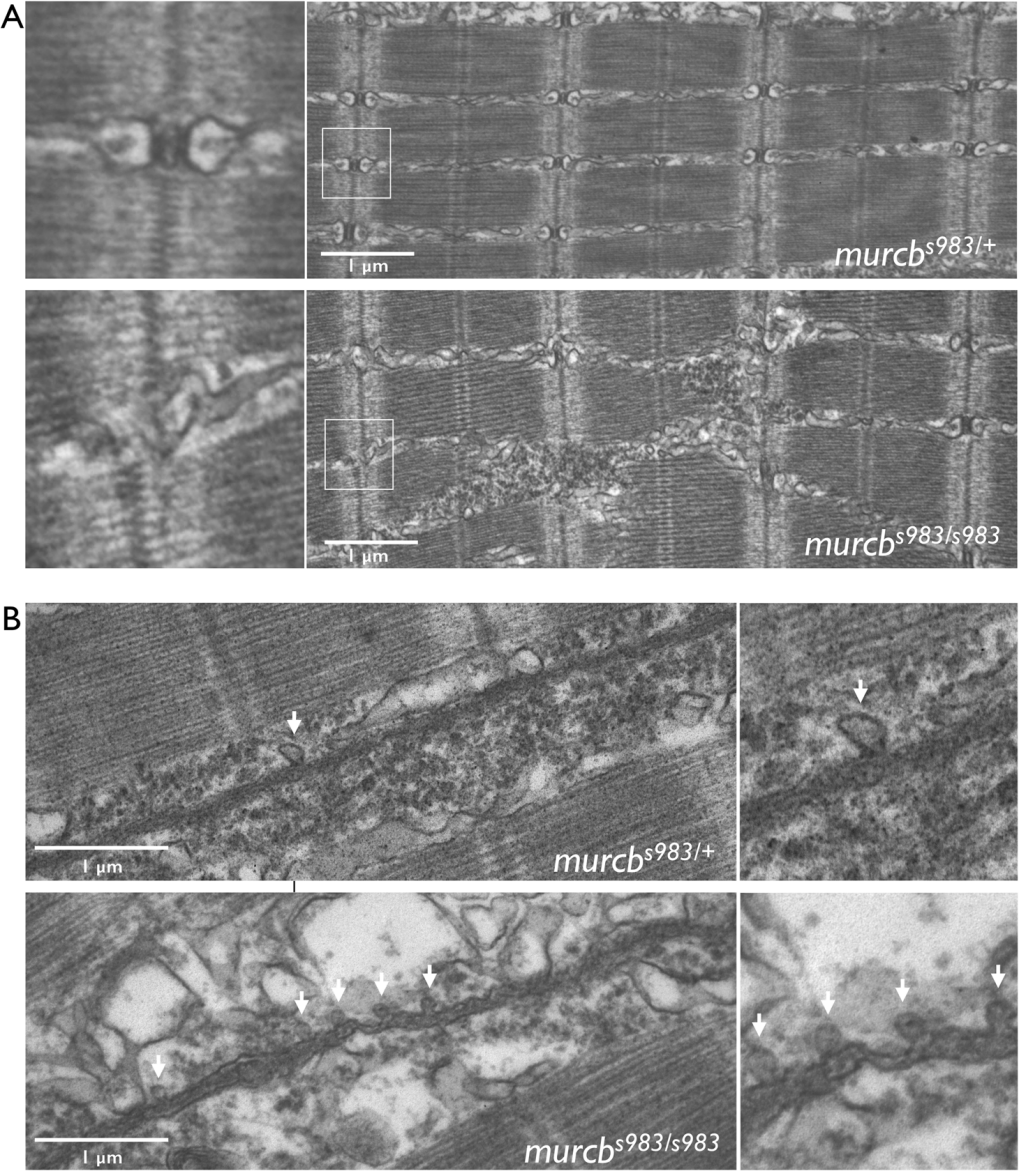
Ultra-structural analysis of Cavin4b/Murcb deficient skeletal muscle. A. Representative electron micrographs of *murcb*^*s983/+*^ and *murcb*^*s983/s983*^ zebrafish at 80 hpf. T-tubule triad structures are indicated with a box and enlargements of this region are shown on the left. B. Representative electron micrographs of *murcb*^*s983/+*^ and *murcb*^*s983/s983*^ zebrafish at 80 hpf. Caveloae are indicated by arrows. Enlargements from the micrographs are shown on the right.

Because voltage-gated ion channels are critical to excitation-contraction coupling, we assessed the ultrastructure of the skeletal muscle in *murcb* mutant larvae to determine whether there were T-tubule abnormalities. Electron microscopy (EM) performed on 80 hpf larvae revealed that Cavin4b/Murcb deficiency was associated with a disruption of T-tubule development and maturation (Fig 6A). Quantification of the electron micrograph data showed that sibling controls had 88.3% intact T-tubule triad structures while Cavin4b/Murcb deficient larvae had 10% intact triads (S2 Table). Additionally, we found an increased number of caveolae in *murcb*^*s983/s983*^ larvae at 80 hpf compared with sibling controls (Fig 6B).

### Cavin4b/Murcb is required for the localization of Caveolin proteins and Erk signaling *in vivo*

**Fig 7:**
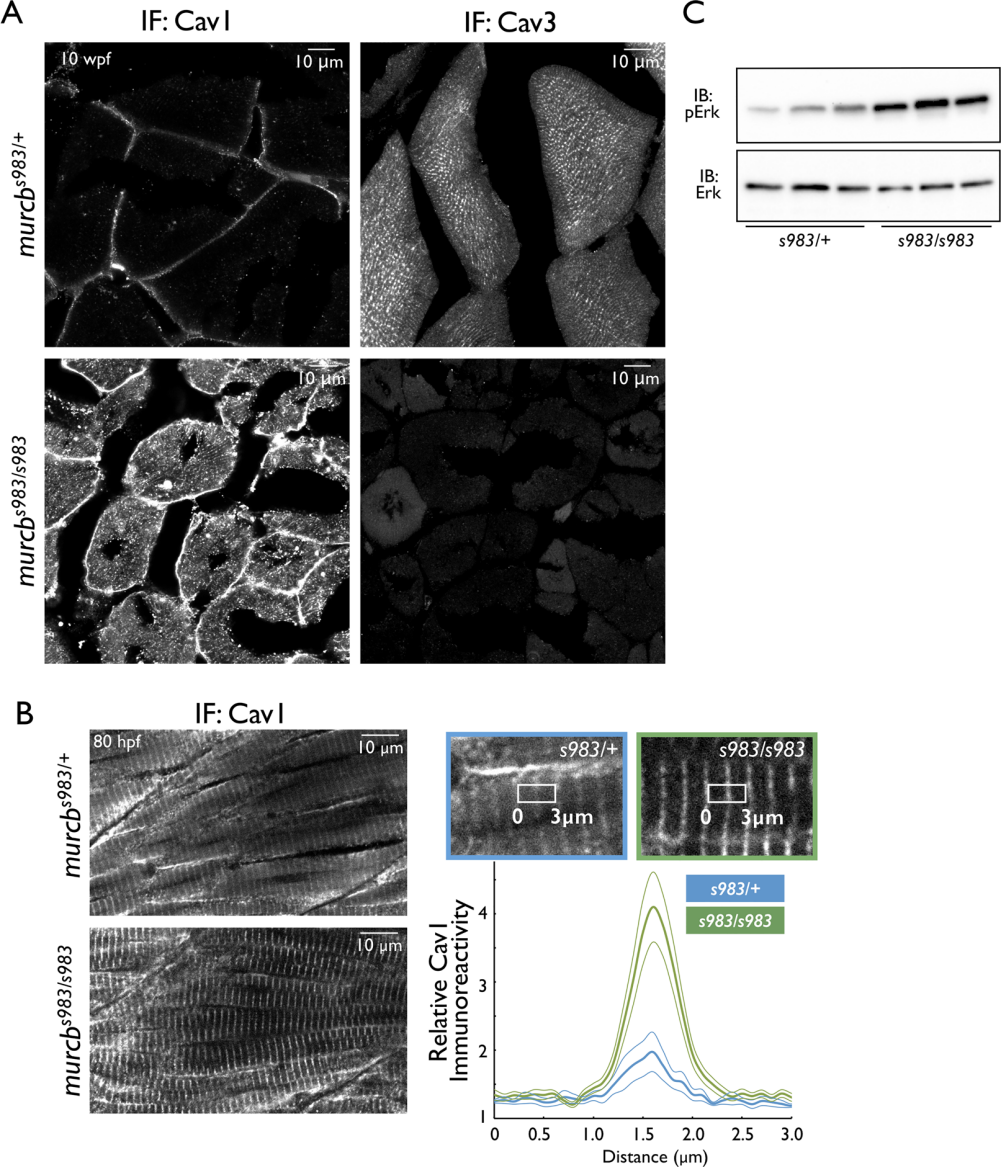
Caveolin expression and growth factor signaling in Cavin4b/Murcb deficient zebrafish. A. Representative confocal micrographs of Cav1 and Cav3 immunostaining of 10 μm transverse sections prepared from the trunk of 10 wpf *murcb*^*s983/+*^ and *murcb*^*s983/s983*^ zebrafish. B. Representative confocal micrographs of Cav1 whole mount immunostaining in *murcb*^*s983/+*^ and *murcb*^*s983/s983*^ zebrafish at 80 hpf. Mean relative fluorescence profiles from at least 6 larvae for each genotype. Pixel intensity was measured across a 3 μm segment covering a single striation and plotted relative to the background intensity between stria. SEM is represented by thin lines. C. Immunoblot of phospho-Thr202/Tyr204-Erk1/2 and total Erk1/2 from skeletal muscle prepared from 3 *murcb*^*s983/+*^ and 3 *murcb*^*s983/s983*^ 10 wpf zebrafish.

Because of the role of Cavin4/Murc in the localization of Caveolin proteins, we examined the localization of Cav1 in transverse sections from 10 wpf fish and found increased and mislocalized anti-Cav1 immunoreactivity (Fig 7A). In sections from heterozygous siblings, Cav1 was predominantly localized to the surface of skeletal muscle fibers while in *murcb*^*s983/s983*^ skeletal muscle, there appeared to be additional Cav1 staining in the internal, putative membrane structures. In contrast to what has been reported in mammals [21,29], we found that Cav3 does not localize to the surface of skeletal muscle fibers in 10 wpf zebrafish, but rather appears to be associated with internal structures and not with Cavin4/Murc (S5 Fig). Interestingly, Cavin4b/Murcb is nonetheless required for Cav3 localization, which is disrupted in *murcb*^*s983/s983*^ samples (Fig 7A).

In order to determine if Murcb has an effect on Cav1 localization in larvae, we performed whole mount antibody staining on larvae at 80 hpf. We found that Cav1 localization was altered in *murcb*^*s983/s983*^ larvae compared with heterozygous siblings with increased localization in a striated pattern (Fig 7B). To profile the staining of the striations, we measured the pixel density relative to the background of confocal images along a 3 μm distance perpendicular to the striations. We then plotted the pixel intensity on the y-axis versus the distance across the image on the x-axis and found that compared to heterozygous siblings, *murcb*^*s983/s983*^ larvae have a higher Cav1 antibody reactivity in these striations.

Because caveolae are known to regulate growth factor signaling, we also assayed phosphorylated Erk1/2 in Cavin4b/Murcb deficient fish. We performed immunoblotting of skeletal muscle lysates from 3 *murcb*^*s983/s983*^ zebrafish and 3 *murcb*^*s983/+*^ siblings at 10 wpf and found in each mutant that phospho-Erk1/2 was increased relative to total Erk1/2 (Fig 7C).

## Discussion

Here, we have shown a requirement for Cavin4b/Murcb in the development and function of skeletal muscle. Additionally, we have shown that Cavin4b/Murcb is necessary *in vivo* for caveolae function, Caveolin-1 and -3 localization, and Erk signaling in skeletal muscle. Our analyses of a targeted *murcb* mutation reveal the importance of caveolae for the formation of T-tubules during myogenesis and suggest an additional etiology for muscular dystrophy.

The complex membrane structures necessary for skeletal muscle function, such as the T-tubule network, require specialized membrane microdomains and their associated proteins for formation and function. Disruption of these proteins can lead to human disease. The nature of the Cavin4b/Murcb dependent muscle pathology described here is likely due to defects in the formation of T-tubules as evidenced by immature triad structures at 80 hpf. Caveolae are known to be abundant in T-tubules during development [22]; Cav3 is required for T-tubule shape and organization, and *Cav3* mutant mice exhibit similar myopathologies as patients with type 1C limb-girdle MD [23]. Skeletal muscle fibers lacking a functional T-tubule network likely have calcium flux and excitation-contraction coupling deficiencies that could result in ion channel gene expression changes initially, and subsequently result in muscle wasting [6].

Several genetic models have yielded insights into the pathology of other types of MD. For example ENU-induced mutations in zebrafish laminin β2 (*lamb2*/*softy*) cause embryonic muscle degeneration due to loss of the extracellular matrix supporting the sarcolemma during skeletal muscle contraction [36]. Zebrafish *dystrophin* (*sap/dmd*) mutants display skeletal muscle fiber detachment and membrane damage resulting in uptake of Evans blue dye [37]. These models of MD present phenotypes early in development and are therefore identifiable in a forward genetic screen. In contrast *cavin4b/murcb*, a much smaller gene than *lamb2* or *dmd*, is less likely to be mutagenized by random means and is more likely to be determined to be phenotypically normal prior to 5 dpf. Therefore, a targeted genetic approach was critical to understand the function of *murcb* in zebrafish. The pathomechanics of Cavin4b/Murcb deficiency appear to be rather different than those of dystrophin deficiency as *dystrophin* mutant larvae show muscle detachment by 48 hpf and die around 31 dpf [37]. Therefore, *cavin4b/murcb* mutants represent a distinct tool to better understand processes such as T-tubulogenesis and how caveolinopathies lead to skeletal muscle diseases.

Mutations in other Cavin genes have been linked to skeletal muscle disease. Mutations in human CAVIN1 (PTRF) have been reported to cause MD and generalized lipodystrophy, defects that are likely secondary to caveolae disruption [38]. Patients in this study had histopathologies in common with MD such as variable skeletal muscle fiber size, while other phenotypic changes were not consistently present, suggesting the influence of genetic modifiers. It will be interesting to examine CAVIN4 localization and expression in patients with CAVIN1 mutations, as there appears to be interdependence in their expression and regulation. Indeed, it was recently shown that in *Cavin1* KO mice, CAV1 protein expression was reduced and CAVIN4 expression was not detectable [39]. This reduction in CAVIN4 expression was evident in both skeletal muscle and heart, and suggests that a homeostatic feedback mechanism exists that either stabilizes a Cavin hetero-oligomeric complex or regulates transactivation. Kovtun et al. have recently characterized Cavin interactions, finding that CAVIN1 can bind to other Cavin family members as well as homo-oligomerize [31]. This interaction is mediated through the Helical Region (HR1), which forms a trimeric coiled coil. Cavin oligomerization seems to be organ-specific [39], and may depend on interactions with CAVIN4 or CAV3, which have restricted expression patterns. In human skeletal muscle, CAVIN4 expression correlates very highly with mosaic expression of CAV3 [29].

While the function of Cavin4/Murc remains poorly understood, a role in signal transduction has emerged in recent studies. In cultured cardiomyocytes, hypoxia induces Cavin4/Murc expression in an Erk-dependent manner [40]. A Cavin4/Cav3 complex co-localizes with α1-adrenergic receptors (α1-AR) in cardiomyocytes and α1-AR-induced cardiac hypertrophy is attenuated in *Cavin4/Murc* mutant mice [30].

Apart from a direct upstream effect of Cavin4/Murc on Erk signaling, one explanation for the observed increase in phosphorylated Erk in Cavin4/Murc deficient zebrafish skeletal muscle is chronic injury [41]. Satellite cell exhaustion from continual repair is thought to contribute to the pathology of MD. Interestingly, *Cavin4/Murc*, but not *Cav1* or *Cav3*, is highly upregulated in “alert” satellite cells [42] (see http://www.ncbi.nlm.nih.gov/geo/query/acc.cgi?acc=GSE55490) raising the possibility that Cavin proteins are necessary for muscle membrane structures as well as for repair, perhaps through a role in cell fusion [43,44]. Further examination of the Cavin4/Murc KO mouse, which in contrast to zebrafish has not been reported to have a skeletal muscle phenotype [30], should be directed toward muscle injury and repair.

Caveolar proteins have been linked to cardiomyopathies as well as MD [45–47]. In mouse, mutations in *Cav3* result in cardiomyopathy characterized by myocyte hypertrophy, dilation of the heart chambers, and a reduction in fractional shortening [48]. Given the interdependence between Cavin and Caveolin protein expression and distribution, the role of Cavin4 in cardiomyopathy should be studied further. *CAVIN4* mutations have previously been linked to Dilated Cardiomyopathy (DCM). Rodriguez et al found 6 heterozygous mutations (N128K, R140W, L153P, S307T, P324L, and S364L) in patients with DCM but none in patients with hypertrophic cardiomyopathy or in healthy controls[49]. Our Cavin4b/Murcb deficient fish line will be a useful tool to determine whether these mutations in human *CAVIN4* indeed result in a loss of protein function. As there is an overexpression phenotype for Cavin4/Murc reported in mouse [30], the cloning of the zebrafish *cavin4b/murcb* promoter/enhancer will likely be necessary for rescue experiments with the human mutant alleles.

The pathomechanics of the various forms of MD have been of great interest due to the severity of the disease, limited treatment options, and the difficulty of implementing gene therapy [4]. Due to the accessibility of developing skeletal muscle in zebrafish larvae, zebrafish models of MD can be used to test how aberrant calcium flux, T-tubule development, and growth factor signaling can impact progressive skeletal muscle disease. A targeted mutation approach has revealed that Cavin4b/Murcb is necessary for the development of the T-tubule network during embryonic myogenesis and the proper localization of Caveolin-1 and -3 in adult muscle. These changes are associated with reduced skeletal muscle fiber size and uniformity, fibrosis, and reduced muscle function, suggesting that CAVIN4 may be important to the etiology of MD early in the development of skeletal muscle.

## Materials and Methods

### Immunoblotting, immunostaining, histology and cell death visualization

Antibodies used for immunoblotting were against Erk1/2 (Cell Signaling, 9102), phosphoThr202/Tyr204-Erk1/2 (Cell Signaling, 4370), Caveolin-1 (BD Transduction Labs, 610059), Caveolin-3 (BD Transduction Labs, 610420), and Cavin4/Murc (Sigma-Aldrich, HPA021021). Whole mount antibody and phalloidin stainings were performed on larvae fixed with 4% paraformaldehyde, 4% sucrose, 77 mM Na_2_HPO_4_, 22.6 mM NaH_2_PO_4_, 120 μM CaCl_2_ pH 7.35 overnight at 4°C, permeabilized with PBS, 0.5% Triton X-100 for 3 hours at room temperature, and stained with Alexa-488 conjugated phalloidin (Cytoskeleton, PHDG1) at a 1:100 dilution in PBS, 0.5% Triton X-100. Whole mount immunofluorescence staining was performed on fixed larvae that were dehydrated in 4 steps to 70% ethanol in PBS and stored at 4°C. Prior to staining, larvae were rehydrated in 4 steps to 1X PBS, digested with proteinase K at 30 μg/ml for 30 minutes at room temperature and blocked with PBS, 5% normal goat serum (NGS), 0.5% Triton X-100 and incubated with indicated antibodies diluted 1:100 in PBS, 5% NGS, 0.5% Triton X-100 overnight at 4°C. Larvae were then washed 4 times with PBS with 0.5% Triton X-100, incubated with secondary antibodies, mounted in agarose, and imaged on an LSM700 confocal microscope (Zeiss). H&E and immunofluorescence stainings were performed on trunk tissue dissected from adult fish and fixed in 4% paraformaldehyde, 4% sucrose, 77 mM Na_2_HPO_4_, 22.6 mM NaH_2_PO_4_, 120 μM CaCl_2_ pH 7.35 overnight at 4°C, transferred to PBS with 20% sucrose for 24 hours and then frozen in OCT for cryosectioning. 10 μm transverse cryosections were stored at -80°C prior to staining. Sections were briefly dried, then permeabilized with PBS with 0.5% Triton X-100. Alexa-488 conjugated phalloidin was diluted 1:100 in PBS with 0.5% Triton X-100 and incubated for 3 hours at room temperature followed by 5 washes with PBS with 0.5% Triton X-100. Indicated antibodies were diluted in PBS, 5% NGS, 0.5% Triton X-100 and incubated overnight at 4°C. Sections were then washed 4 times with PBS with 0.5% Triton X-100 and incubated with Alexa-488 or Alexa-568 conjugated secondary antibodies and imaged on an LSM700 confocal microscope. Presynaptic structures were visualized in 80 hpf fish that were fixed in 4% paraformaldehyde for 4 hours at 4°C, washed 4 times with PBS, and permeabilized with protease K (10 μg/ml) for 30 minutes at room temperature. Larvae were then washed in PBS, 0.5% Triton X-100, 1% DMSO (PBT-DMSO) before blocking overnight at 4°C in PBT-DMSO with 1% BSA and 2% goat serum. Antibodies against Synaptic Vesicle 2 (SV2, Developmental Studies Hybridoma Bank) were diluted 1:20 in blocking solution and incubated overnight at 4°C. After washing with PBT-DMSO, larvae were incubated with Alexa-568 conjugated donkey anti-mouse antibodies (Molecular Probes) for 2 hours at room temperature. Larvae were then washed, mounted, and imaged on an LSM 780 confocal microscope (Zeiss). Postsynaptic structures were visualized in 80 hpf fish that were fixed in 4% paraformaldehyde for 4 hours at 4°C, washed 4 times with PBS, permeabilized with 0.2% collagenase P (Roche, 11213805001) for 50 minutes then washed 4 times with PBS. Embryos and larvae were incubated with 20 μg/ml Tetramethylrodhamine (TRITC) conjugated bungarotoxin (Sigma Aldrich, T0195) for 30 min at room temperature. Larvae were then washed, mounted, and imaged on an LSM 780 confocal microscope. Acridine orange (Sigma, 235474) staining was used to identify apoptosis in live larvae at 80 hpf. Larvae were incubated in a solution of 10 μg/ml acridine orange for 30 minutes, then washed with PBS three times and imaged through a Fluorescein-Isothiocyanate (FITC) filter using an LSM780 confocal microscope.

### Mass spectrometry

#### Sample preparation

3 dpf zebrafish larvae were genotyped and trunk tissue was homogenized in SDS lysis buffer (4% SDS in 100 mM Tris/HCl pH 7.6). For complete cell lysis, samples were heated at 95°C for 10 minutes. The homogenate was spun at 16,000 g for ten minutes and protein concentration was estimated using the DC protein assay (Biorad). Lysates were precipitated for one hour using 4 volumes of ice-cold acetone. The protein pellet was dissolved in 6 M urea, 2 M thiourea, 10 mM HEPES, pH 8 and subjected to digestion in solution. Next, protein disulfide bonds were reduced with 10 mM dithiothreitol (DTT) and alkylated with 55 mM iodoacetamide. In-solution digestion was done with Lys-C (Wako) (protein to Lys-C ratio = 100:1) overnight at room temperature. The resulting peptide mixtures were concentrated and desalted using the Stop and Go Extraction (STAGE) technique [50].

Liquid Chromatography and Mass Spectrometry: A binary buffer system consisting of buffer A (0.1% formic acid or 0.5% acetic acid) and buffer B (80% Acetonitrile, 0.1% formic acid or 0.5% acetic acid) was used for peptide separation on an Easy nano-flow UHPLC system (Thermo Fisher Scientific, Odense Denmark). This system was coupled via a nano electrospray ionization source to the QExactive mass spectrometer. Peptide elution from the in-house packed 50 cm columns (1.8 μm Beads, 75 μm ID, Dr. Maisch, Germany) was achieved by increasing the relative amount of B from 7% to 38% in a linear shape within 240 min at a column temperature of 45°C, followed by 5 min at 95% B and gradients were completed by a re-equilibration time of 5 min to 5% B. Acquisition of MS spectra in a mass range of 350–1650 m/z was done using an AGC target of 3E6 at a resolution of 70000 (200 m/z). The instrument worked in a data dependent mode, isolation of the ten most intense peaks for HCD fragmentation (25 collision energy) in a 100–1650 m/z mass range. The AGC target was set to 5E5 combined with a resolution of 35000 at 200 m/z, while a maximum injection time of 60 ms was used.

#### Data analysis

Complete set of raw files was processed using MaxQuant (1.5.2.8) and the implemented Andromeda search engine. For protein assignment ESI-MS/MS fragmentation spectra were correlated to the Uniprot zebrafish database (*Danio rerio*, 40368 entries) including a list of contaminants. A maximum of two missed cleavages and a mass tolerance of 4.5 ppm and 7 ppm for MS/MS first and main search were set, respectively. Carbamidomethyl at cysteine residues was defined as a fixed modification and oxidation at methionine and acetylation at the N-terminus of proteins were set as variable modifications. A minimal peptide length of 7 amino acids after Lys-C specificity for protein assignment and for quantification a minimal ratio count of 2 was required. Further, only unique peptides were used for quantification and the FDR was controlled by the revert algorithm in MaxQuant at the peptide and protein level. Data visualization was performed using the statistical environment R and MaxQuant output text files were filtered for contaminants and reverse entries. Gene Ontology annotation based on Uniprot identifiers was carried out in the Perseus software (Max Planck Institute, Martinsried).

### Membrane integrity assay

Evans Blue Dye (Sigma Aldrich, E2129) was dissolved in egg water at 0.1% (w/v). 4 dfp 1-phenyl-2-thiourea (PTU)-treated larvae were transferred to a nylon mesh basket and submerged in dye for 60 minutes. The basket was removed from the dye solution, washed with egg water, and larvae were transferred to a fresh dish for imaging and genotyping.

### Microarray and PANTHER analysis

RNA and gDNA were extracted from individual larvae at 72 hpf using Trizol (Life Technologies). Following genotyping, the aqueous phases from at least 10 Trizol extractions were combined by genotype and purified over microspin columns (ZymoResearch). RNA expression from heterozygous and mutant larvae was analyzed by single-color microarray (8x60K Zebrafish Array XS, Oaklabs, Germany). The arrays were imaged using a SureScan Microarray Scanner and Agilent’s Feature Extraction software version 11 was used to read and process the TIFF images. Data were normalized by using the ranked median quantiles [51]. Microarray data were archived in the NCBI database (GSE70858).

PANTHER (v 9.0, release 20140124) database protein class statistical overrepresentation test (release 20141219, with Bonferroni correction) was performed on a list of genes that expressed at a ratio of greater than or equal to 1.5 times or less than or equal to 0.7 times the expression found in heterozygous *murcb versus* homozygous *murcb* larvae and having a normalized signal with a value greater than 50. 1309 differentially expressed genes were identified in the database. 25708 genes comprise the reference set for *Danio rerio*.

### Swimming analysis

Swimming speed and acceleration were measured in 10-week-old fish that were transferred to a 40 cm by 40 cm tray containing system water above which a digital camera was mounted. Movies were recorded following the startle response and frame-by-frame location within the tray over time was determined using PhysMo V2 software (http://physmo.sf.net). Distance calibration was made using a ruler submerged in the tray. Measurements of swimming larvae were performed in a similar manner in a 10 cm dish. Deformed fish were not used in the swimming analysis. Statistical significance was determined using a two-tailed unequal variance *t*-test.

### TALEN mutagenesis, genotyping and RT-PCR

TALENs were designed against *murcb* using the TALEN Targeter 2.0 software [33,34]. The plasmid kit used for generation of TALENs was a gift from Daniel Voytas and Adam Bogdanove (Addgene kit # 1000000024). Capped mRNA encoding the TALEN was generated using the mMESSAGE mMACHINE SP6 Transcription Kit (Life Technologies, AM1340) and 200 pg of each arm were injected into one-cell stage embryos. TALENs were tested by HRMA analysis on an amplicon from gDNA harvested from individual embryos at 24 hpf. Injected F0 siblings from functional TALENs were raised to adulthood and outcrossed to identify founders. F1 fish were then raised to adulthood and gDNA was isolated from the caudal fin to identify F1 heterozygous fish by HRMA. PCR products from the HRMA were cloned into pGEM-T Easy (Promega) for sequencing. gDNA was isolated by digesting individual embryos or clipped fins in proteinase K at 65°C for 1 hour with occasional vortexing followed by inactivation at 95°C for 10 minutes. For screening of founders, HRMA primers were designed to amplify between 80 and 100 nucleotides. Upon identification of a suitable allele, HRMA primers were redesigned to amplify a region immediately surrounding the mutation (Forward: 5’-TCTCTGCTGGAGAGGGTGTC, Reverse: 5’-CTGGCAGGCCTGAACATTGT, Annealing temperature: 65°C). RT-PCR primers for *murca* were: Forward: 5’- TGTGATCTATCAGGGTGAAAAAG, Reverse: 5’- TGGAGAACGCAGCCTTGATGTTC. RT-PCR primers for *murcb* were: Forward: 5’- GTCATCTACCAGGGAGATAATGAG, Reverse: 5’- GTCATGTTCTCGCGTGAGAAGGTC.

### Transmission electron microscopy

Zebrafish larvae were fixed with 3% glutaraldehyde (Merck), followed by 4% OsO4 in 0.1 mmol/L sodium cacodylate buffer (pH 7.4). After dehydration in ethanol and propylene oxide, they were embedded in Epon as described. Semi-thin (1 μm) sections were stained with toluidine blue and viewed in a Leica DM microscope. Ultrathin sections were stained with uranyl acetate and lead citrate and viewed and photographically recorded under a Philips CM 10 electron microscope.

### Ethics statement

Animal studies were carried out following guidelines and standard procedures set forth by the Regierungspräsidium Gießen Dezernat V 54 (Protocol No. B2/342).

## Supporting Information

S1 Fig Murc conservation and expression in zebrafish. A. Protein alignment of zebrafish Murca and Murcb with human, mouse, rat, cow, and *Xenopus* orthologs. B. Zebrafish *murc* genes share a similar intron-exon structure with human *MURC* having two exons and a single intron. White boxes represent UTRs. C. Phylogenetic analysis of zebrafish Murca and Murcb. D. Whole mount *in situ* hybridization of *murca* mRNA in zebrafish embryos at 48 hpf. Top: lateral view. Bottom left: magnified lateral view. Bottom right: magnified dorsal view.
(TIFF)

S2 Fig Mass Spectrometry of lysates generated from a pool of genotyped larval trunk tissue. A. Total ion chromatogram of an in-solution digest from *murcb*^*s983/+*^ and *murcb*^*s983/s983*^ larvae. Arrow at 72.64 minutes retention time points to the peptide mass of 631.35 m/z (see arrow in B). B. MS spectra from *murcb*^*s983/+*^ and *murcb*^*s983/s983*^ samples.
(TIFF)

S3 Fig Analysis of Cavin4b/Murcb deficient zebrafish. A. Evans blue dye assay of membrane integrity. The top panel is an injured somite and is shown as a positive control. No significant changes were observed between mutants and heterozygous controls. B. Representative maximal projection confocal images from live whole mount acridine orange staining of *murcb*^*s983/+*^ and *murcb*^*s983/s983*^ zebrafish trunk at 80 hpf. Arrows point to DNA fragmentation. Dotted lines outline the larvae. No significant changes were observed between mutants and heterozygous controls. C. RT-PCR analysis of *murca* and *murcb* mRNA from *murcb*^*s983/+*^ and *murcb*^*s983/s983*^ larvae at 72 hpf. No significant changes were observed between mutants and heterozygous controls.
(TIFF)

S4 Fig Neuromuscular junction analysis of Cavin4b/Murcb deficient zebrafish. Representative confocal maximal projections of whole mount Synaptic Vesicle glycoprotein 2 (SV2, left) and α-bungarotoxin (α-BTX, right) staining of the trunk of 80 hpf *murcb*^*s983/+*^ and *murcb*^*s983/s983*^ zebrafish. SV2 immunofluorescence was used to visualize presynaptic structures; α-BTX staining was used to visualize postsynaptic structures. Synaptic punctae per somite were quantified using ImageJ (dotplot, bottom).
(TIFF)

S5 Fig Immunofluorescence localization of Cav3 and Murc in adult skeletal muscle. Confocal micrographs of transverse sections prepared from 10 wpf *murcb*^*s983/+*^ zebrafish and stained with anti-Murc (red) and anti-Cav3 (green). Merged views are shown on the right and below.
(TIFF)

S1 Table Label-free quantification (LFQ) of peptides from Murcb deficient larvae and sibling controls. (DOCX)

S2 Table Quantification of intact triads from electron micrographs of Cavin4b/Murcb deficient and sibling zebrafish larvae. (DOCX)
