# Supplementary figures and images for: Cavin4b/Murcb Is Required for Skeletal Muscle Development and Function in Zebrafish
Source: PLoS Genet. 2016 Jun 13;12(6):e1006099. doi: 10.1371/journal.pgen.1006099 (PMC4905656; doi:10.1371/journal.pgen.1006099)

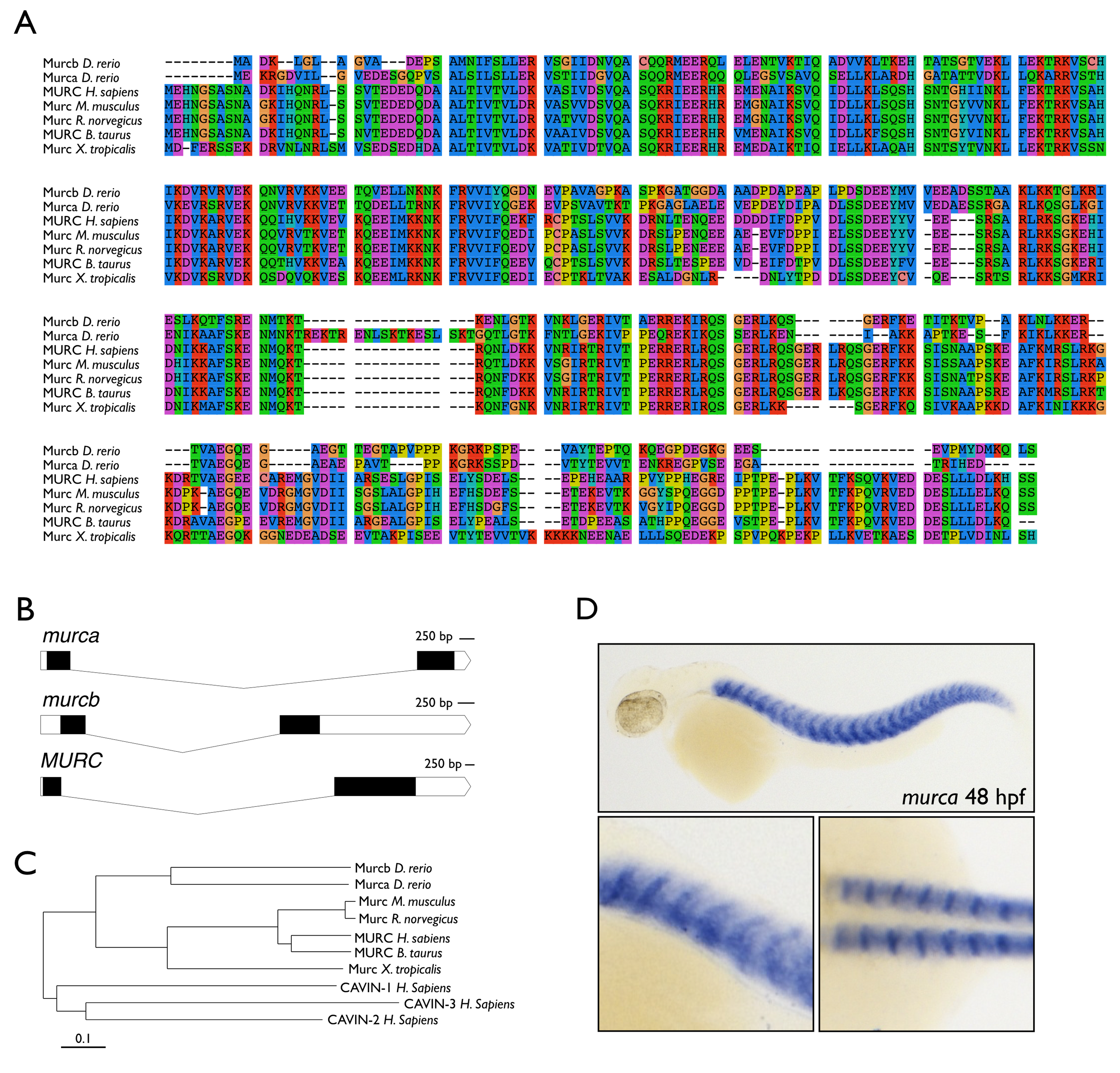

Supplement: S1 Fig — A. Protein alignment of zebrafish Murca and Murcb with human, mouse, rat, cow, and Xenopus orthologs. B. Zebrafish murc genes share a similar intron-exon structure with human MURC having two exons and a single intron. White boxes represent UTRs. C. Phylogenetic analysis of zebrafish Murca and Murcb. D. Whole mount in situ hybridization of murca mRNA in zebrafish embryos at 48 hpf. Top: lateral view. Bottom left: magnified lateral view. Bottom right: magnified dorsal view. (TIFF) [file pgen.1006099.s001.tiff]

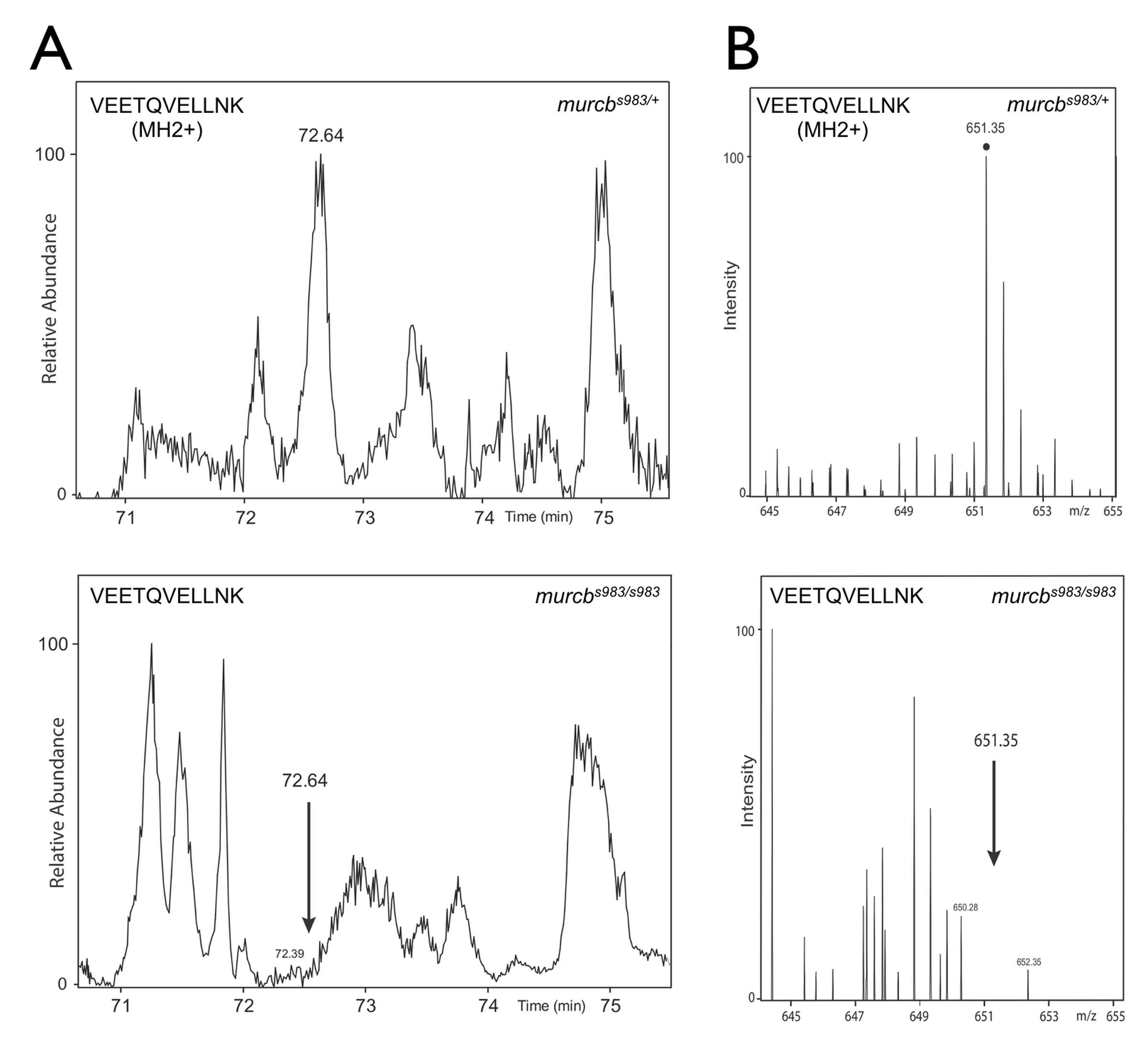

Supplement: S2 Fig — A. Total ion chromatogram of an in-solution digest from murcbs983/+ and murcbs983/s983 larvae. Arrow at 72.64 minutes retention time points to the peptide mass of 631.35 m/z (see arrow in B). B. MS spectra from murcbs983/+ and murcbs983/s983 samples. (TIFF) [file pgen.1006099.s002.tiff]

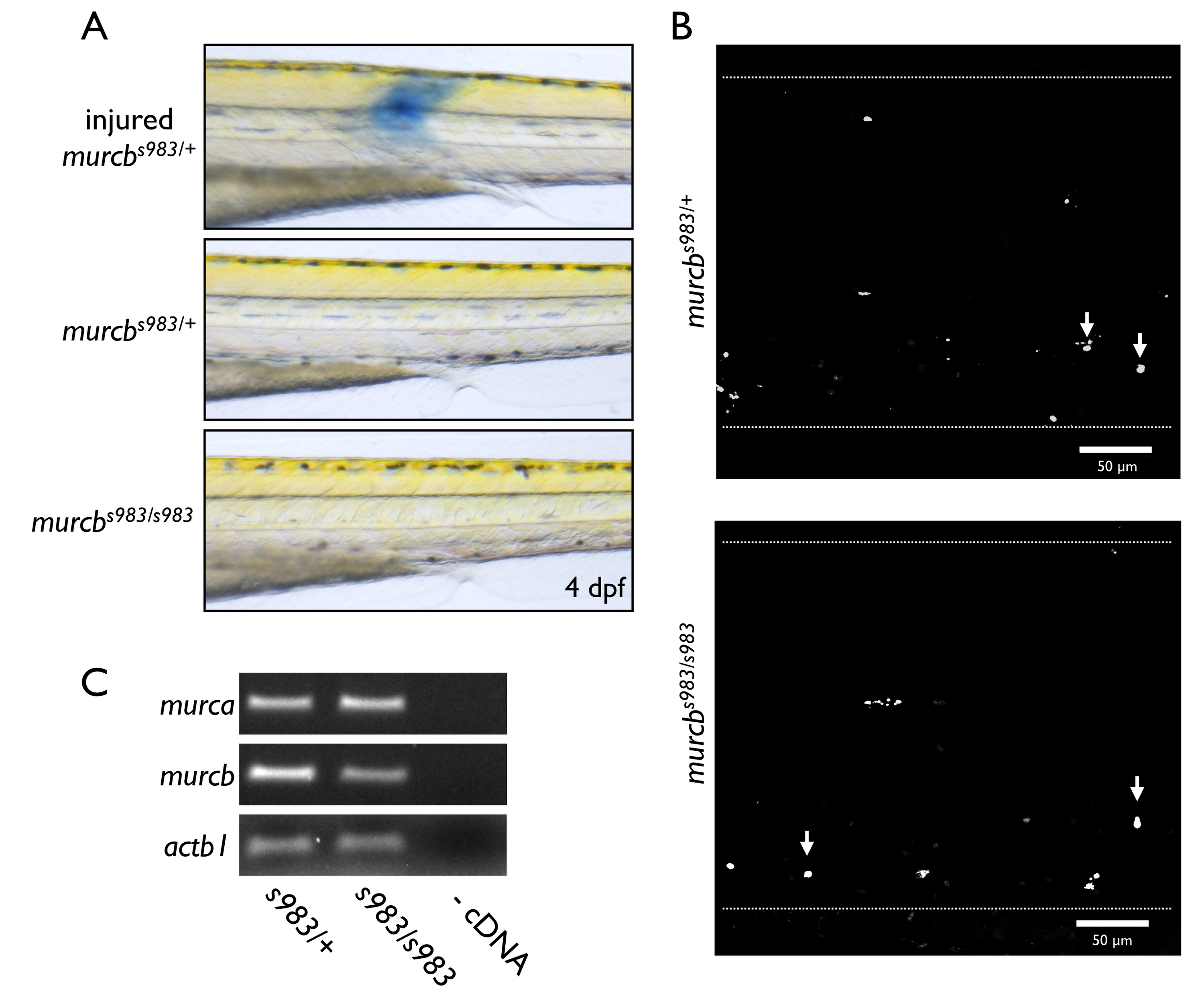

Supplement: S3 Fig — A. Evans blue dye assay of membrane integrity. The top panel is an injured somite and is shown as a positive control. No significant changes were observed between mutants and heterozygous controls. B. Representative maximal projection confocal images from live whole mount acridine orange staining of murcbs983/+ and murcbs983/s983 zebrafish trunk at 80 hpf. Arrows point to DNA fragmentation. Dotted lines outline the larvae. No significant changes were observed between mutants and heterozygous controls. C. RT-PCR analysis of murca and murcb mRNA from murcbs983/+ and murcbs983/s983 larvae at 72 hpf. No significant changes were observed between mutants and heterozygous controls. (TIFF) [file pgen.1006099.s003.tiff]

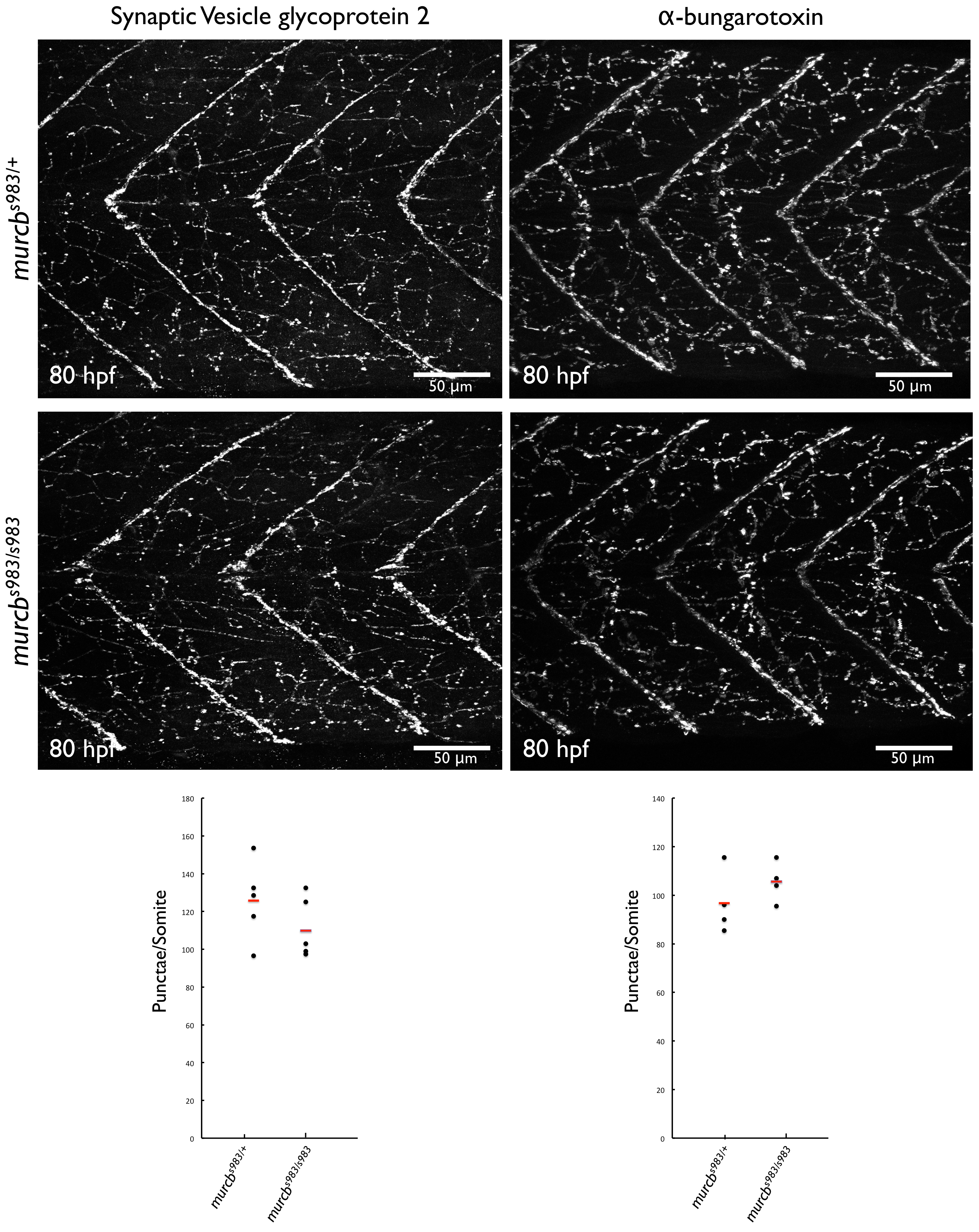

Supplement: S4 Fig — Representative confocal maximal projections of whole mount Synaptic Vesicle glycoprotein 2 (SV2, left) and α-bungarotoxin (α-BTX, right) staining of the trunk of 80 hpf murcbs983/+ and murcbs983/s983 zebrafish. SV2 immunofluorescence was used to visualize presynaptic structures; α-BTX staining was used to visualize postsynaptic structures. Synaptic punctae per somite were quantified using ImageJ (dotplot, bottom). (TIFF) [file pgen.1006099.s004.tiff]

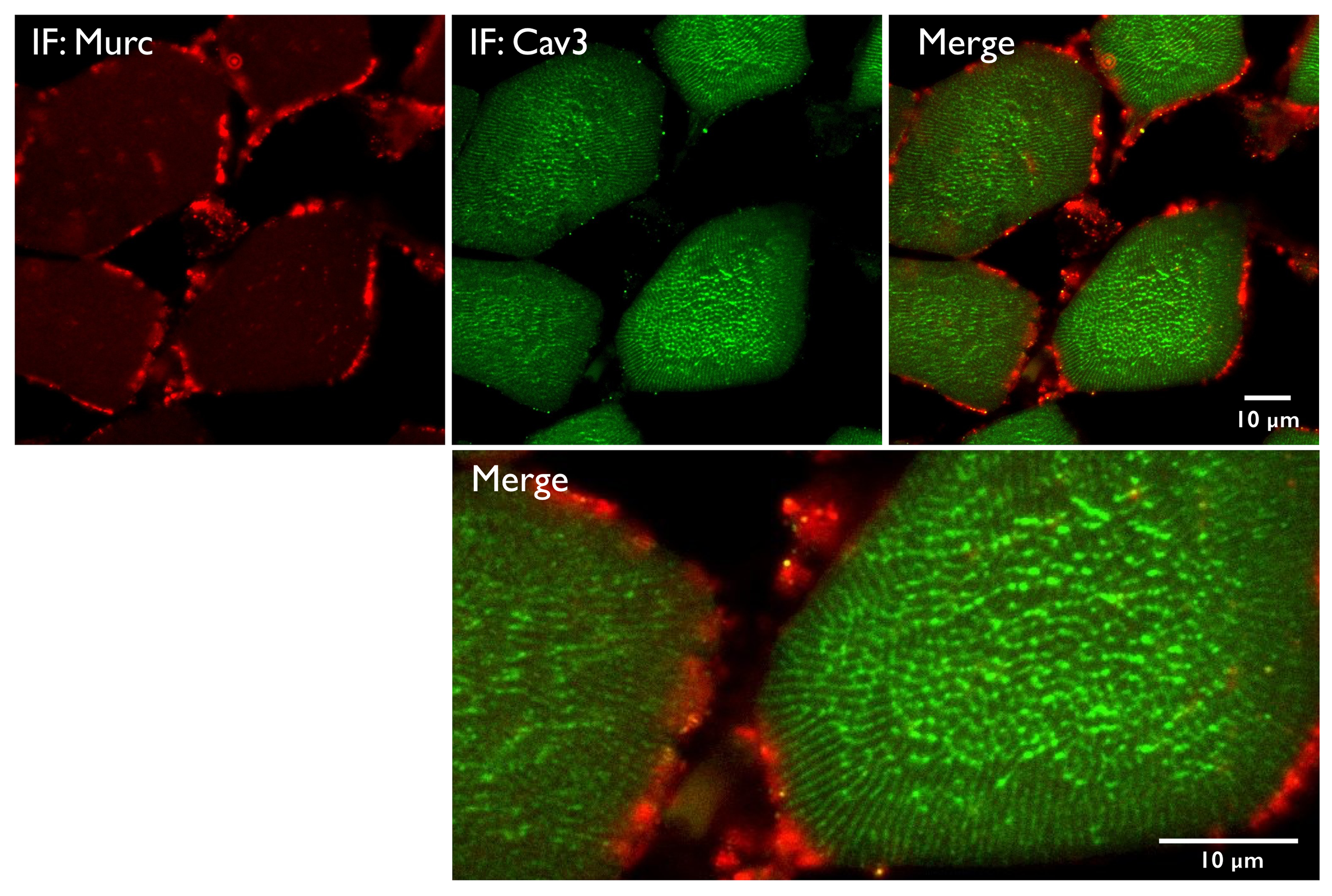

Supplement: S5 Fig — Confocal micrographs of transverse sections prepared from 10 wpf murcbs983/+ zebrafish and stained with anti-Murc (red) and anti-Cav3 (green). Merged views are shown on the right and below. (TIFF) [file pgen.1006099.s005.tiff]
