# Supplementary material for: Cavin4b/Murcb Is Required for Skeletal Muscle Development and Function in Zebrafish
Source: PLoS Genet. 2016 Jun 13;12(6):e1006099. doi: 10.1371/journal.pgen.1006099 (PMC4905656; doi:10.1371/journal.pgen.1006099)
Supplement: S1 Table — (DOCX) [file pgen.1006099.s006.docx]

| **Table S1. Label-free quantification (LFQ) of peptides from Murcb deficient larvae and sibling controls.** | | | | |
| --- | --- | --- | --- | --- |
|  |  |  |  |  |
| Peptide | LFQ Intensity *s983/+* | LFQ Intensity *s983/s983* | Position | Mass/Charge |
| KVEETQVELLNK | 8.18E+06 | ND | aa102-113 | 715.4 |
| TVAEGQEGAEGTTEGTAPVPPPK | 7.03E+06 | ND | aa257-279 | 1112.54 |
| VEETQVELLNK | 1.16E+07 | ND | aa103-113 | 651.35 |
| VSGIIDNVQACQQR | 1.40E+07 | ND | aa26-39 | 878.47 |
