# Supplementary material for: Cavin4b/Murcb Is Required for Skeletal Muscle Development and Function in Zebrafish
Source: PLoS Genet. 2016 Jun 13;12(6):e1006099. doi: 10.1371/journal.pgen.1006099 (PMC4905656; doi:10.1371/journal.pgen.1006099)
Supplement: S2 Table — (DOCX) [file pgen.1006099.s007.docx]

| **Table S2. Quantification of intact triads from electron micrographs of Cavin4b/Murcb deficient and sibling zebrafish larvae.** | | | |
| --- | --- | --- | --- |
| Animal ID | Muscle area (μm^2^) | Percent intact triads |  |
| *s983/+* Nr. 1 | 6722 | 84.3 |  |
| *s983/+* Nr. 2 | 7290 | 97.7 |  |
| *s983/+* Nr. 3 | 7787 | 83.3 |  |
| *s983/+* Nr. 4 | 4773 | 90.9 |  |
| *s983/+* Nr. 5 | 10514 | 85.0 |  |
| *s983/s983* Nr. 1 | 7743 | 15.5 |  |
| *s983/s983* Nr. 2 | 10122 | 9.6 |  |
| *s983/s983* Nr. 3 | 8354 | 11.8 |  |
| *s983/s983* Nr. 4 | 5888 | 8.3 |  |
| *s983/s983* Nr. 5 | 10768 | 4.7 |  |
